# Is there a link between heart rate variability and cognitive decline? A cross-sectional study on patients with mild cognitive impairment and cognitively healthy controls

**DOI:** 10.1055/s-0042-1758862

**Published:** 2023-03-14

**Authors:** Bernhard Grässler, Milos Dordevic, Sabine Darius, Fabian Herold, Giuseppe Forte, Corinna Langhans, Nicole Halfpaap, Patrick Müller, Wenzel Glanz, Estélio Henrique Martin Dantas, Irina Böckelmann, Notger Müller, Anita Hökelmann

**Affiliations:** 1 Otto von Guericke University, Institute of Sport Science, Magdeburg, Germany.; 2 German Center for Neurodegenerative Diseases, Neuroprotection Research Group, Magdeburg, Germany.; 3 Otto von Guericke University, Department of Neurology, Medical Faculty, Magdeburg, Germany.; 4 University of Potsdam, Faculty of Health Sciences, Degenerative and Chronic Diseases Research Group, Movement, Potsdam, Germany.; 5 Otto von Guericke University, Faculty of Medicine, Department of Occupational Medicine, Magdeburg, Germany.; 6 Sapienza Università di Roma, Dipartimento di Psicologia, Rome, Italy.; 7 IRCCS Fondazione Santa Lucia, Action and Body Lab, Rome, Italy.; 8 Otto- von- Guericke University Magdeburg, Institute of Cognitive Neurology and Dementia Research, Magdeburg, Germany.; 9 German Research Group Neuroprotection Center for Neurodegenerative Diseases, Magdeburg, Germany.; 10 Universidade Federal do Estado do Rio de Janeiro, Programa de Pós- Graduação em Enfermagem e Biociências, Rio de Janeiro RJ, Brazil.; 11 Universidade Tiradentes, Programa de Pós- Graduação em Saúde e Ambiente, Aracaju SE, Brazil.; 12 Center Research Group Neuroprotection for Behavioral Brain Sciences, Magdeburg, Germany.

**Keywords:** Heart Rate, Cognitive Dysfunction, Cognition, Autonomic Nervous System Diseases, Frequência Cardíaca, Disfunção Cognitiva, Cognição, Doenças do Sistema Nervoso Autônomo

## Abstract

**Background** Given that, up to date, there is no effective strategy to treat dementia, a timely start of interventions in a prodromal stage such as mild cognitive impairment (MCI) is considered an important option to lower the overall societal burden. Although autonomic functions have been related to cognitive performance, both aspects have rarely been studied simultaneously in MCI.

**Objective** The aim of the present study was to investigate cardiac autonomic control in older adults with and without MCI.

**Methods** Cardiac autonomic control was assessed by means of heart rate variability (HRV) at resting state and during cognitive tasks in 22 older adults with MCI and 29 healthy controls (HCs). Resting HRV measurement was performed for 5 minutes during a sitting position. Afterwards, participants performed three PC-based tasks to probe performance in executive functions and language abilities (i.e., Stroop, N-back, and a verbal fluency task).

**Results** Participants with MCI showed a significant reduction of HRV in the frequency-domain (high frequency power) and nonlinear indices (SD2, D2, and DFA1) during resting state compared to HCs. Older individuals with MCI exhibited decreases in RMSSD and increases in DFA1 from resting state to Stroop and N-back tasks, reflecting strong vagal withdrawal, while this parameter remained stable in HCs.

**Conclusion** The results support the presence of autonomic dysfunction at the early stage of cognitive impairment. Heart rate variability could help in the prediction of cognitive decline as a noninvasive biomarker or as a tool to monitor the effectiveness of therapy and prevention of neurodegenerative diseases.

## Resumo

**Antecedentes** Como não existe até o momento uma estratégia eficaz para tratar a demência de comprometimento cognitivo leve (MCI, na sigla em inglês), as intervenções em um estágio prodrômico são consideradas uma opção. Embora as funções autonômicas tenham sido relacionadas ao desempenho cognitivo, ambos os aspectos raramente foram estudados simultaneamente no MCI.

**Objetivo** Investigar o controle autonômico cardíaco em idosos com e sem MCI.

**Métodos** O controle autonômico cardíaco foi avaliado por meio da variabilidade da frequência cardíaca (HRV, na sigla em inglês) em repouso e durante tarefas cognitivas, em 22 idosos com MCI e 29 controles saudáveis (HCs, na sigla em inglês). A medida da HRV de repouso foi realizada por 5 minutos na posição sentada. Os participantes realizaram três tarefas executadas em computador para testar o desempenho em funções executivas e habilidades de linguagem (o teste de cores e palavras - Stroop, Tarefa N-back auditiva e uma tarefa de fluência verbal).

**Resultados** Em pacientes com MCI, observou-se uma redução significativa da HRV no domínio da frequência (potência de alta frequência) e índices não lineares (SD2, D2 e DFA1) durante o estado de repouso em comparação com os HCs. Indivíduos mais velhos com MCI exibiram diminuições em RMSSD e aumentos em DFA1 do estado de repouso para Stroop e tarefas N-back, refletindo forte recessão vagal, enquanto este parâmetro permaneceu estável em HC.

**Conclusão** Observou-se disfunção autonômica na fase inicial da neurodegeneração. A HRV pode ajudar na previsão do declínio cognitivo, como um biomarcador não invasivo, ou como uma ferramenta para monitorar a eficácia da terapia e prevenção de doenças neurodegenerativas.

## INTRODUCTION

A healthy human heart shows beat-to-beat variations in the electrocardiogram (ECG). 1 These oscillations of the heart rate are described as heart rate variability (HRV) and are the result of the complex interaction between the activity of the sympathetic and parasympathetic nervous systems. 2 3 Heart rate variability has been recognized as an important marker of mental and physical health, especially to assess the risk of developing cardiovascular diseases. 2 On the one hand, chronic stress, inflammation, reduced regulatory capacity, obesity, smoking, and cardiovascular diseases are associated with reduced indices of HRV. 4 On the other hand, an optimal HRV indicates a healthy organism, adaptability, and well-being. 3 5 Hence, HRV is a promising marker to detect pathological states. 1

A link between autonomic and cognitive processes is supported by several studies demonstrating a positive relationship between cognitive functioning and HRV. 6 7 8 However, there are only a few studies using markers of the autonomic nervous system (ANS), such as HRV, to differentiate between normal age-related cognitive decline and mild cognitive impairment (MCI). Moreover, the results of these studies are rather inconclusive. While some authors reported a reduced HRV in older adults with MCI, 9 others found only a slightly lower HRV, 10 or even observed no differences between MCI and healthy controls (HCs). 11 Given that MCI constitutes a prodromal stage of cognitive impairment that is characterized by deficits in memory and/or in other cognitive domains such as executive functions while normal functioning of daily activities are preserved, 12 it can be very challenging to diagnose and distinguish MCI from older adults with normal, age-related cognitive decline using only behavioral indicators. Based on the evidence (i) that older individuals with MCI have a higher risk of developing dementia compared with older adults with normal cognition 13 and (ii) that autonomic dysfunction is a vascular risk factor accelerating cognitive decline, 9 the evaluation of cardiac autonomic control through HRV might provide a better understanding of the physiological mechanisms stimulating the transition from healthy aging to MCI. In this context, it seems promising to quantify HRV at rest and during cognitive tasks in older adults with MCI and HCs, as measurement during the execution of a cognitive task allows to assess the response of the ANS to the mental load. 14 A decrease in vagally mediated HRV from resting state to a cognitive task has been termed “vagal withdrawal” 15 . Regarding this reactivity of the ANS, one can expect higher vagal withdrawal in older adults with MCI compared with HCs to compensate their cognitive deficits.

The aim of the present study was to evaluate differences in ANS function between older adults with MCI and HCs by measuring HRV at resting state and during cognitive tasks. We hypothesized (i) a higher HRV in HCs compared with MCI at resting state, and (ii) a stronger vagal withdrawal in MCI in response to the cognitive tasks compared to HCs.

## METHODS

### Participants

**Figure 1:**
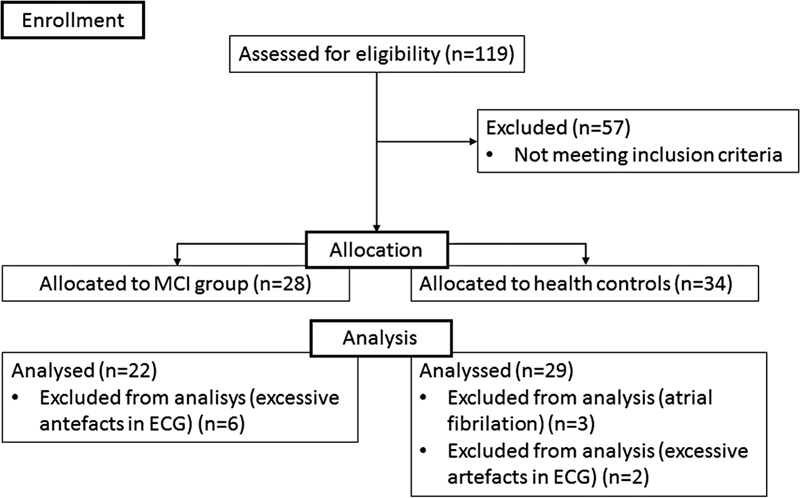
Study flow diagram.

One-hundred and nineteen participants from Magdeburg and surroundings (Germany) were initially recruited through advertisements with flyers, posters, and newspapers and using existing databases looking for people with subjective memory impairments. A flow diagram of the sample selection process is shown in Figure 1 . The required sample size was determined on the basis of prior research examining Stroop performance in older adults with MCI and HCs. 16 According to the means and standard deviations (SDs) of the error rate in the Stroop incongruent task, a sample size of 17 participants per group was calculated to achieve a power of 0.80, and an effect size of 1.00 with an alpha level of *p* < 0.05. Considering at least 20 participants per group provided a sufficient buffer for possible dropouts.

The participants were screened for eligibility based on the following inclusion criteria: 50 to 80 years old; native German-speaking; living and able to manage everyday activities independently. Participants with other neurological diseases (i.e., epilepsy, multiple sclerosis), severe cardiac diseases (i.e., severe cardiac insufficiency, cardiac pacemaker, valvular defect (stenosis and insufficiency of aortic or mitral valve), arterial hypertension, cardiac arrhythmias, atrial fibrillation), mental diseases (i.e., schizophrenia or depression), orthopedic diseases (i.e., bone fracture in the last 6 months, symptomatic slipped disc), muscular diseases (i.e., myositis, tendovaginitis), severe endocrinologic diseases (i.e., manifest hypothyroidism or hyperthyroidism, insulin dependent type 2 diabetes), injury or surgery in the last 6 months, using illegal intoxicants or alcohol abuse, uncorrected eyesight or hearing, anamnestic known color blindness or red-green weakness, pregnancy or breastfeeding, using neuroleptics, narcotic analgesics, benzodiazepines, or psychoactive medications were excluded from the study. Participants who met our criteria were administered the Consortium to Establish a Registry for Alzheimer's Disease (CERAD) plus test battery (including the Mini Mental State exam [MMSE]) 17 to evaluate their cognitive state. Participants who scored 1.5 z-scores below the age- and education-adjusted reference sample in at least 1 subtest were referred to experienced neurologists. Mild cognitive impairment diagnosis was made in accordance with Petersen et al. 18 and participants were allocated to the MCI (of all types) or to the healthy group. After being informed on possible risks and benefits associated with the study, the participants provided written informed consent. The present study was approved by the local ethics committee (reference: 83/19) and is in accordance with the latest version of the Declaration of Helsinki. The study was registered in ClinicalTrials.gov (NCT04427436) on the June 10, 2020.

### Testing procedure

Prior to the neurophysiological assessment, general data, such as body weight (in kg), height (in cm), body mass index (BMI), and level of education were recorded. During the neurophysiological assessment, physiological data were collected during resting state and while participants performed cognitive tasks. The resting state measurement lasted for 5 minutes with a preceding stabilization period of 5 minutes. Participants were instructed to refrain from intense physical training and drinking alcohol 24 hours before the measurement. Drinking caffeinated drinks, smoking, and eating were not allowed two hours before the experiment to limit potential acute effects on HRV. A detailed description of the measurement procedure can be found in the study protocol. 19

### Cognitive tasks

Before the experiment started, the participants were briefed on the task instructions and experimental design. Prior to the tasks, the participants were adequately familiarized with the tests by completing a sufficient number of practice trials. All tasks were administered via computer using the software Presentation (Neurobehavioral Systems Inc, San Francisco, CA, USA). On-task HRV was calculated by averaging the mean values of all the three task blocks in each condition.

#### Stroop

The Stroop task is a widely used task to assess inhibition and cognitive control, 20 which are crucial for completion of complex cognitive tasks and everyday activities. 16 It included three experimental conditions in a fixed order to ensure an even increase in task difficulty. In each block, 20 color-words were consecutively presented on a computer screen. Four different color-words appeared: “RED,” “GREEN,” “BLUE,” or “YELLOW” in German language. Each color had a corresponding button. In the congruent condition, the meaning of the color-word and the ink color matched. In the incongruent condition, the color-word was printed in an incongruent ink color, e.g., the word “RED” was presented in blue color. Participants were instructed to identify the color of the word by pressing the appropriate button and ignoring the meaning of the word. Participants were advised to react as fast and as correct as possible. The third condition was a mixed block design with congruent stimuli within an incongruent condition.

#### N-back

The N-back task is a frequently used task that probes working memory capacity. 21 Three levels of difficulty were used: 0-, 1-, and 2-back. Each condition consisted of 3 blocks with 20 stimuli. Single-digit numbers were presented consecutively on the screen. In the 0-back condition, participants were instructed to press the target button when the number “7” appeared. In the 1-back condition, the target was any number identical to the number immediately presented before. In the 2-back condition, the target was any number identical to the second-last number presented before. Participants were instructed to react as fast and as correct as possible. The performance of the Stroop and N-back tasks were operationalized by the percentage of errors and mean reaction time of correct responses. Wrong responses, no responses, or responses below a reaction time of 100 milliseconds were considered as errors.

#### Verbal fluency test

**Figure 2:**
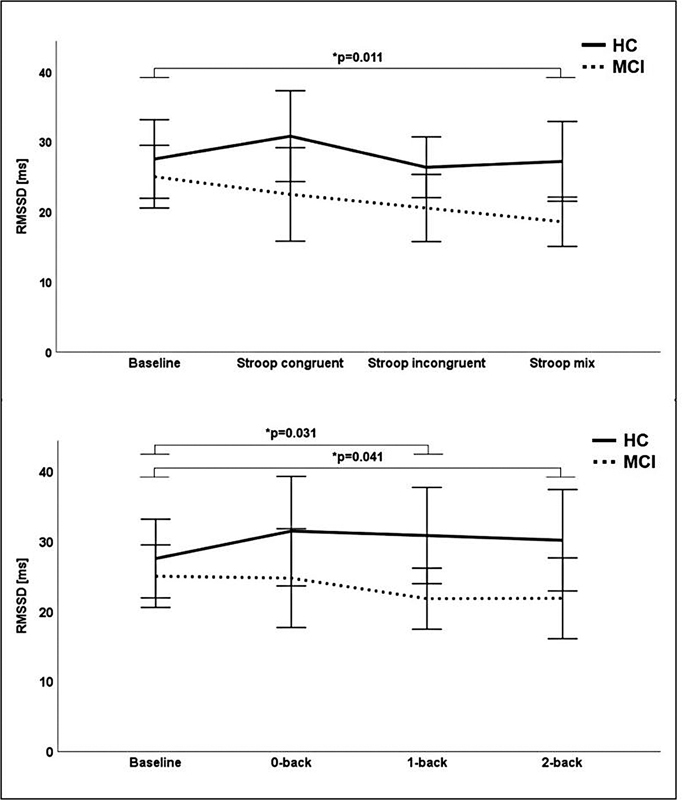
Means of RMSSD during baseline, Stroop, and N-back of MCI (dashed line) and HCs (continuous line). Error bars indicate ± 2 standard error of mean; significance level ( *p* ) indicates group x condition interaction effects from ANCOVA with age and level of education as covariates. Abbreviations: HC, healthy controls; MCI, mild cognitive impairment; RMSSD, root mean square of successive differences of NN intervals.

Verbal fluency has been shown to be impaired in neurodegenerative disorders such as MCI, 22 and thus was assessed by a verbal fluency task based on the “Regensburger Wortflüssigkeitstest”. 23 The verbal fluency test (VFT) consisted of two conditions ( Figure 2 ). In the phonological condition, participants were instructed to pronounce as many German words (nouns, verbs, adjectives) as possible beginning with a specific letter (“A,” “F,” “P”). In the semantic condition, participants were instructed to pronounce words belonging to a specific category (“forenames,” “fruits,” “flowers”). The number of correctly recited words was used as measure of performance.

### ECG recording and processing

Electrocardiogram data were collected using a three-channel Holter-ECG (Medilog AR12plus, Schiller Medizintechnik GmbH, Baar, Switzerland). The raw ECG data were checked automatically and visually by a healthcare professional for pathological abnormalities. Heart rate variability analysis was performed using Kubios premium 3.3 (University of Kuopio, Finland).

Heart rate variability analysis comprised traditional and most often used time-domain (SD of all NN intervals in milliseconds [SDNN]; root mean square of successive differences of NN intervals in milliseconds [RMSSD]) and frequency-domain (power in low frequency range [0.04–0.15 Hz] [LF]; power in high frequency range [0.15–0.4 Hz] [HF]; LF/HF; total power [TP]) parameters. 1 As nonlinear parameters also provide valuable information on health status but are rarely used in the literature, three nonlinear parameters were analyzed as well: SD along the line of identity in the Poincaré plot (SD2); correlation dimension (D2); and detrended fluctuation analysis (DFA1). Since a recording time of at least 5 minutes is required for frequency-domain parameters and SD2, these parameters were analyzed only for the resting state measurement. 24 Time-domain parameters as well as D2 and DFA1 were analyzed for the periods of resting state and cognitive tasks because these parameters are just as valid for short-term recordings < 5 minutes. 25 26 27

According to recent recommendations, participants were sitting with their knees bent at a 90° angle, their hands on their thighs, eyes closed, and were advised to relax and breath normally. 15 After the baseline recording, participants completed three cognitive tasks. To avoid artefacts, they were asked not to move or talk during the measurement.

### Statistical analysis

The normality of the data was assessed by using the Shapiro-Wilk test. The chi-squared test was used to detect differences between groups in their gender distribution. Demographic, behavioral, and baseline HRV data were compared between the groups through the independent samples t-test. Effect size d was calculated and rated as small (d > 0.2), medium (d < 0.5), and large (d < 0.8). 28 An analysis of covariance (ANCOVA) with age and level of education as covariates was performed to investigate effects of group and reactivity of HRV to mental load, with condition (resting state versus on-task) as the within-subjects factor and group (HC versus MCI) as the between-subjects factor. All statistical tests were performed using IBM SPSS Statistics for Windows version 26.0 (IBM Corp., Armonk, NY, USA). For all analyses, the level of significance was set at *p* < 0.05. We reported ANCOVA effect size as partial eta squared (η ^2^ ). The effect size magnitude was rated as follows: ≥ 0.01 small, ≥ 0.059 medium, and ≥ 0.138 large effects. 28

## RESULTS

### Demographic characteristics

Participants with > 5% artefacts or outliers ( *n* = 8) and participants with atrial fibrillation were excluded from the analysis ( *n* = 8). The final sample comprised 22 MCI and 29 HCs. Older adults with MCI and HCs were well matched with no significant differences in age, gender distribution, BMI, and level of education ( Table 1 ). Participants with MCI had significantly lower z-scores in the CERAD test battery ( *p* < 0.001; d = 1.834) and lower MMSE scores compared with HCs ( *p* < 0.001; d = 3.092).

**Table 1:** Demographic data of the participants

Variable	MCI ( *n* = 22)	HC ( *n* = 29)	*p-value*	Effect size d
Age (years old)	70.19 ± 6.04	68.18 ± 6.73	0.276 ^a^	0.312
Male/female ( *n* )	10/12	6/23	0.074 ^b^	0.548
BMI (kg/m ^2^ )	25.21 ± 2.05	24.95 ± 3.40	0.734 ^a^	0.090
Education (years)	14.18 ± 1.99	14.62 ± 1.80	0.414 ^a^	0.234
CERAD (z-score)	-0.62 ± 0.36	0.03 ± 0.35	< 0.001*** ^a^	1.834
MMSE (score)	26.41 ± 0.67	28.72 ± 0.80	< 0.001*** ^a^	3.092
Reaction time Stroop congruent (ms)	1.041 ± 0.150	0.909 ± 0.172	0.006** ^a^	0.727
Error rate Stroop congruent (%)	2.58 ± 2.80	1.49 ± 1.36	0.107 ^a^	0.519
Reaction time Stroop incongruent (ms)	1.638 ± 0.554	1.128 ± 0.262	0.001** ^a^	1.234
Error rate Stroop incongruent (%)	22.15 ± 29.12	2.59 ± 2.73	0.005** ^a^	1.020
Reaction time Stroop mix (ms)	1.312 ± 0.334	1.019 ± 0.256	0.001** ^a^	0.906
Error rate Stroop mix (%)	8.11 ± 15.03	1.55 ± 2.09	0.055 ^a^	0.658
Reaction time 0-back (ms)	0.489 ± 0.059	0.460 ± 0.060	0.095 ^a^	0.487
Error rate 0-back (%)	0.15 ± 0.49	0.00 ± 0.00	0.162 ^a^	0.468
Reaction time 1-back (ms)	0.525 ± 0.101	0.471 ± 0.085	0.044* ^a^	0.586
Error rate 1-back (%)	0.15 ± 0.49	0.06 ± 0.31	0.406 ^a^	0.227
Reaction time 2-back (ms)	0.637 ± 0.133	0.561 ± 0.117	0.035* ^a^	0.612
Error rate 2-back (%)	4.55 ± 5.02	3.05 ± 3.40	0.381 ^a^	0.360
A… ( *n* )	6.23 ± 1.69	6.83 ± 1.91	0.249 ^a^	0.330
F… ( *n* )	7.05 ± 1.94	8.48 ± 2.84	0.037* ^a^	0.521
P… ( *n* )	5.55 ± 2.26	6.76 ± 2.37	0.071 ^a^	0.521
Sum letter ( *n* )	18,.82 ± 3.50	22.07 ± 5.66	0.011 * ^a^	0.691
Forenames ( *n* )	11.73 ± 3.01	16.14 ± 3.86	< 0.001*** ^a^	1.130
Fruits ( *n* )	9.27 ± 2.39	10.69 ± 2.35	0.039* ^a^	0.540
Flowers ( *n* )	7.45 ± 2.09	9.52 ± 2.65	0.004** ^a^	0.767
Sum category ( *n* )	28.45 ± 5.09	36.34 ± 6.79	< 0.001*** ^a^	1.315

Abbreviations: BMI, body mass index; CERAD, Consortium to Establish a Registry for Alzheimer Disease; HC, healthy controls; MCI, mild cognitive impairment; MMSE, Mini Mental State Exam; ms, millisecond.

Note: continuous variables expressed as mean ± standard deviation, categorial variables as *n* ; ^a^ : Student's t-test; ^b^ : Chi-squared test; * *p* < 0.05; ** *p* < 0.01; *** *p* < 0.001.

### Cognitive tasks data

Healthy controls displayed better performance on all cognitive tasks compared with MCI participants, who responded less accurately, had longer reaction times, and produced fewer words than HCs ( Table 1 ). Significant differences were detected for error rate in Stroop incongruent ( *p =* 0.005; d = 1.020), reaction time in Stroop congruent ( *p =* 0.006; d = 0.727), incongruent ( *p =* 0.001; d = 1.234), and mix ( *p* = 0.001; d = 0.906), as well as for reaction time in 1-back ( *p* = 0.044; d = 0.586) and 2-back ( *p* = 0.035; d = 0.612). Moreover, HCs pronounced significantly more words beginning with F ( *p* = 0.037; d = 0.521), more names ( *p* < 0.001; d = 1.130), fruits ( *p* = 0.039; d = 0.540), and flowers ( *p* = 0.004; d = 0.767). Finally, HCs pronounced significantly more words beginning with a specific letter ( *p* = 0.011; d = 0.691) and belonging to a specific category ( *p* < 0.001; d = 1.315).

### HRV at resting state

Significant differences between HCs and MCI were observed for HF ( *p* = 0.048; d = 0.519) and TP ( *p =* 0.030; d = 0.577) ( Table 2 ). Standard deviation along the line of identity in the Poincaré plot ( *p =* 0.017; d = 0.697), D2 ( *p =* 0.030; d = 0.593), and DFA1 ( *p =* 0.017; d = 0.682) showed significant differences as well. Medium effect sizes for these group differences were observed.

**Table 2:** Between-group comparisons for HRV values at baseline using t-test

Variable	MCI ( *n* = 22)	HC ( *n* = 29)	*p-value*	Effect size d
mHR (bpm)	65.96 ± 7.82	63.98 ± 7.16	0.352	0.266
SDNN (ms)	21.62 ± 8.50	28.56 ± 14.49	0.051	0.565
RMSSD (ms)	25.05 ± 10.49	27.57 ± 15.13	0.507	0.189
LF (ms ^2^ )	214.07 ± 312.20	542.91 ± 800.37	0.075	0.515
HF (ms ^2^ )	162.00 ± 107.28	283.70 ± 295.74	0.048*	0.519
TP (ms ^2^ )	428.14 ± 424.21	906.58 ± 1033.20	0.030*	0.577
LF/HF	1.51 ± 1.55	2.58 ± 3.32	0.171	0.395
SD2 (ms)	24.24 ± 11.14	35.03 ± 18.07	0.017*	0.697
D2	0.47 ± 0.92	1.20 ± 1.42	0.030*	0.593
DFA1	0.84 ± 0.33	1.05 ± 0.29	0.017*	0.682

Abbreviations: bpm, beats per minute; D2, correlation dimension; DFA1, detrended fluctuation analysis; HC, healthy controls; HF, high frequency power; LF, low frequency power; MCI, mild cognitive impairment; mHR, mean heart rate; ms, millisecond; RMSSD, root mean square of successive NN interval differences; SDNN, standard deviation of all NN intervals; SD2, standard deviation along the line of identity in the Poincaré plot; TP, total power; VFT, verbal fluency task.

Note: variables expressed as mean ± standard deviation; * *p* < 0.05.

### Main group effects in HRV

Statistical analysis revealed several main group effects after adjusting for age and education for the cognitive tasks. There was a significant effect for SDNN in the condition Stroop mix ( *p =* 0.044; η ^2^ = 0.083), and for D2 in 1-back ( *p =* 0.031; η ^2^ = 0.095) and 2-back ( *p =* 0.030; η ^2^ = 0.096). A significant difference was observed for D2 in the VFT category ( *p =* 0.007; η ^2^ = 0.146).

### Interaction effects in HRV

**Figure 3:**
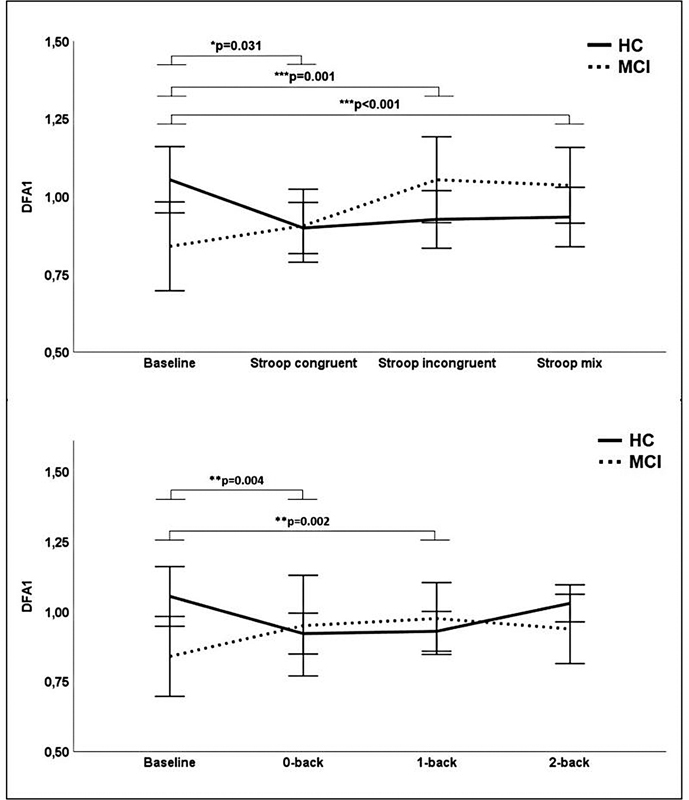
Means of DFA1 during baseline, Stroop, and N-back of MCI (dashed line) and HCs (continuous line). Error bars indicate ± 2 standard error of mean; significance level ( *p* ) indicates group x condition interaction effects from ANCOVA with age and level of education as covariates. Abbreviations: HC, healthy controls; MCI, mild cognitive impairment.

Significant group and condition interaction effects were found for RMSSD ( Figure 2 ) and DFA1 ( Figure 3 ) ( Table 3 ). Significant interaction effects for RMSSD were observed in the conditions Stroop mix ( *p =* 0.011; η ^2^ = 0.131), 1-back ( *p =* 0.031; η ^2^ = 0.095), and 2-back ( *p =* 0.041; η ^2^ = 0.086). Significant effects were also observed for DFA1 in the conditions VFT letter ( *p =* 0.015; η ^2^ = 0.120), VFT category ( *p =* 0.044; η ^2^ = 0.083), Stroop incongruent ( *p =* 0.001; η ^2^ = 0.206), as seen on Figure 3 , Stroop mix (p < 0.001; η ^2^ = 0.236), 0-back ( *p =* 0.004; η ^2^ = 0.160), and 1-back ( *p =* 0.002; η ^2^ = 0.183).

**Table 3:** Interaction effects using ANCOVA with group (MCI versus HC) as between-subjects factor and condition (baseline versus task condition) as within-subject factor, *p-value* and effect size

Condition	Variable	Group difference (95%CI)		
Baseline	mHR (bpm)	1.98 (−2.38, 6.05)		
	SDNN (ms)	−6.94 (−12.82, 1.24)		
	RMSSD (ms)	−2.52 (−9.23, 6,25)		
	D2	−0.73 (−1.25, 0.08)		
	DFA1	−0.21 (−0.40, −0.04)		
**Condition**	**Variable**	**Group difference (95% CI)**	***p-value***	**Effect size η2**
Stroop congruent	mHR (bpm)	1.24 (−3.15, 5.84)	0.653	0.004
	SDNN (ms)	−7.88 (−15.81, 1.13)	0.509	0.009
	RMSSD (ms)	−8.32 (−18.40, 1.29)	0.052	0.078
	D2	−0.48 (−1.10, 0.16)	0.703	0.003
	DFA1	0.01 (−0.08, 0.18)	0.006**	0.150
Stroop incongruent	mHR (bpm)	1.12 (−3.49, 6.01)	0.651	0.004
	SDNN (ms)	−3.04 (−10.02, 5.19)	0.378	0.017
	RMSSD (ms)	−5.82 (−13.18, 0.32)	0.147	0.044
	D2	−0.45 (−0.93, 0.12)	0.526	0.009
	DFA1	0.13 (0.02, 0.33)	0.001**	0.206
Stroop mix	mHR (bpm)	0.91 (−3.26, 5.42)	0.504	0.010
	SDNN (ms)	−8.71 (−15.82, −0.48)	0.409	0.015
	RMSSD (ms)	−8.62 (−16.20, −1.04)	0.011*	0.131
	D2	−0.62 (−1.18, 0.02)	0.981	0.000
	DFA1	0.10 (−0.01, 0.29)	< 0.001***	0.236
0-back	mHR (bpm)	1.24 (−2.56, 5.40)	0.677	0.004
	SDNN (ms)	−7.31 (−15.93, 3.79)	0.925	0.000
	RMSSD (ms)	−6.73 (−17.61, 5.15)	0.225	0.031
	D2	−0.48(−1.00, 0.16)	0.615	0.005
	DFA1	0.03 (−0.10, 0.25)	0.004**	0.160
1-back	mHR (bpm)	0.56 (−3.33, 4.58)	0.249	0.028
	SDNN (ms)	−8.56(−16.11, 0.96)	0.409	0.015
	RMSSD (ms)	−9.02 (−17.75, 0.59)	0.031*	0.095
	D2	−0.55 (−1.09, 0.04)	0.870	0.001
	DFA1	0.05 (−0.07, 0.21)	0.002**	0.183
2-back	mHR (bpm)	0.93 (−3.02, 5.23)	0.565	0.007
	SDNN (ms)	−8.86 (−17.08, 0.33)	0.300	0.023
	RMSSD (ms)	−8.29 (−18.71, 1.65)	0.041*	0.086
	D2	−0.50 (−0.97, −0.01)	0.765	0.002
	DFA1	−0.09 (−0.20, 0.06)	0.106	0.055
VFT letter	mHR (bpm)	−1.23 (−4.62, 3.39)	0.146	0.045
	SDNN (ms)	−2.57 (−9.47, 3.60)	0.398	0.015
	RMSSD (ms)	−1.18 (−8.74, 4.87)	0.900	0.000
	D2	−0.40 (−1.17, 0.30)	0.726	0.003
	DFA1	0.00 (−0.09, 0.13)	0.015*	0.120
VFT category	mHR (bpm)	−1.56 (−5.41, 3.28)	0.111	0.053
	SDNN (ms)	−3.23 (−10.95, 2.64)	0.699	0.003
	RMSSD (ms)	−1.62 (−11.55, 4.79)	0.729	0.003
	D2	−0.67 (−1.44, −0.50)	0.742	0.002
	DFA1	−0.01 (−0.12, 0.16)	0.044*	0.083

Abbreviations: bpm, beats per minute; CI, confidence interval; D2, correlation dimension; DFA1, detrended fluctuation analysis; HC, healthy controls; MCI, mild cognitive impairment; mHR, mean heart rate; ms, millisecond; RMSSD, root mean square of successive NN interval differences; SDNN, standard deviation of all NN intervals; VFT, verbal fluency task.

Note: Baseline is the same for all conditions; group differences expressed as difference between mean values of MCI and HCs (95% confidence interval). * *p* < 0.05; ** *p* < 0.01; *** *p* < 0.001.

## DISCUSSION

The main finding of the present study was that MCI participants demonstrated significant lower HRV compared to HCs at resting state, during Stroop mix, 1- and 2-back as well as VFT category. Analysis of variance (ANOVA) revealed a stronger decline of HRV in MCI compared to HCs from resting state to the cognitive tasks. These findings point to a stronger vagal withdrawal in MCI compared with HCs from resting state to the cognitive tasks imposing a substantial cognitive workload.

The significant difference in HF between MCI and HCs at resting state is in line with a previous study. 9 Other studies showed no differences in the supine position, but a reduced sympathetic activity in response to an orthostatic stress situation suggesting an orthosympathetic dysfunction in MCI participants. 11 29 A strength of our study is the inclusion of three nonlinear HRV parameters, namely SD2, D2, and DFA1, which have been rarely used in psychophysiological studies although they offer new opportunities to monitor cardiac autonomic control and better reflect physiological processes as they are not linear. 30 Additionally, they offer the advantage of being independent of the mean heart rate and are also suitable for short and nonstationary data series. 26 31 In our study, participants with MCI showed significantly lower values compared to HCs, indicating lower heart rate complexity in MCI participants. Specifically, lower values in DFA1 imply greater randomness, and greater unpredictability in heart rate time series. 26 Moreover, nonlinear parameters have been shown to be suitable to predict mortality in postinfarction patients. 32 Therefore, it could be hypothesized that individuals with MCI and reduced DFA1, are at increased risk for cardiovascular diseases.

Based on the vagal tank theory, highlighting the importance of assessing the reactivity of the ANS in psychophysiological research, 15 we assessed HRV at resting state and during cognitive tasks. Heart rate variability in response to cognitive tasks has been explored in younger 33 and older adults, 14 but to the best of our knowledge, there is currently no study in older adults with MCI available. RMSSD decreased in the MCI group from resting state to the Stroop and N-back tasks but remained more stable in HCs. This behavior suggests a stronger vagal withdrawal and a higher mental workload in MCI participants in response to the cognitive load. However, the results are limited by the lack of statistical interaction effects for Stroop congruent, Stroop incongruent, and 0-back. The increase of DFA1 in the MCI group and the decrease in HCs from resting state to the Stroop and N-back tasks support the findings of Young et al., 34 suggesting that nonlinear measures might serve as valuable predictors of complex behavior such as cognitive performance. As stated by Laborde et al., 15 interpretation of reactivity requires more concern than HRV measured at one time point. In the present study, participants with MCI did not show a significant decrease of RMSSD from resting state to the tasks demanding executive functions (i.e., Stroop incongruent and 2-back) although the decrease was more pronounced as compared to HCs. Based on this observation, no clear conclusions can currently be drawn regarding the adaptability of the ANS in MCI participants in response to a higher mental workload imposed by cognitive tasks.

Our findings, in conjunction with the results of Collins et al., 9 suggest that the cardiovagal system is affected at the early stage (e.g., MCI) of cognitive impairment. Several possible mechanisms of autonomic dysfunction and neurodegeneration are discussed in the literature. Firstly, deteriorations in the central autonomic network, whose inputs are responsible for vagally mediated HRV, cause autonomic dysfunction and cognitive deterioration. 35 Secondly, neurochemical changes may also contribute to cognitive deficits and neurodegeneration. 9 Among them are cholinergic deficiency and inhibition of acetylcholine release, which enhances the release of proinflammatory cytokines promoting cognitive decline. 5 Thirdly, an increase in inflammatory markers, such as C-reactive protein, is related to the aggravation of autonomic dysfunction and neurodegeneration. 36 Finally, autonomic dysfunction is associated with blood pressure dysregulation, affecting cerebral perfusion and accelerating cognitive decline. 11 37

Several limitations of the present study should be mentioned. Firstly, the measuring blocks during the cognitive tasks were relatively short. Future studies might consider longer task blocks that can also be used to reveal differences between MCI and HCs being caused by mental fatigue. Secondly, our sample size is relatively small. Furthermore, the sample included a relatively wide age range to capture a broad cross-section of the population, but this might have influenced both HRV and cognition. Thirdly, given the exploratory nature of the present study, we included participants on medications that potentially affect HRV and removed only participants on medications that surely affect HRV and/or cognition (i.e., neuroleptics, narcotic analgesics, benzodiazepines, and psychoactive medications). Testing a sample of participants with no medications would result in a study sample that is not representative of the general population. Finally, no diagnosis, based on functional or structural methods, has been performed to confirm neurodegenerative diseases.

In conclusion, autonomic dysfunction is a central feature in the development of neurodegenerative diseases and may act as a novel biomarker that can predict worsening of cognitive performance. In the present study, participants with MCI and HCs were compared by means of HRV analysis. The findings support the presence of a reduced parasympathetic modulation at resting state and during execution of cognitive tasks in MCI. Deteriorations in the components of the central autonomic network, neurochemical changes, and cerebral hypoperfusion might be possible mechanisms in the development of autonomic dysfunction during neurodegenerative processes. In future studies, additional physiological data (metabolic, electrophysiological, or hormonal) should be assessed, and longitudinal studies are needed to elucidate the causal relationship between autonomic dysfunction and neurodegeneration and to assess the predictive utility of HRV as a supplementary biomarker in the preclinical stage of dementia.
